# Ethical frameworks of informed consent in the age of pediatric precision medicine

**DOI:** 10.1017/pcm.2024.3

**Published:** 2024-05-06

**Authors:** David Chen

**Affiliations:** Temerty Faculty of Medicine, University of Toronto, Toronto, ON, Canada; Department of Medical Oncology, Dana Farber Cancer Institute, Boston, MA, USA

**Keywords:** precision medicine, ethics, consent, assent, pediatric

## Abstract

Precision medicine is an emergent medical paradigm that uses information technology to inform the use of targeted therapies and treatments. One of the first steps of precision medicine involves acquiring the patient’s informed consent to protect their rights to autonomous medical decision-making. In pediatrics, there exists mixed recommendations and guidelines of consent-related practices designed to safeguard pediatric patient interests while protecting their autonomy. Here, we provide a high-level, clinical primer of (1) ethical informed consent frameworks widely used in clinical practice and (2) promising modern adaptations to improve informed consent practices in pediatric precision medicine. Given the rapid scientific advances and adoption of precision medicine, we highlight the dual need to both consider the clinical implementation of consent in pediatric precision medicine workflows as well as build rapport with pediatric patients and their substitute decision-makers working alongside interdisciplinary health teams.

## Impact statement

Precision medicine holds great promise to personalize medical therapies and treatments for each patient in order to improve clinical outcomes of disease. However, there remains hesitation among patients who may be unfamiliar with this promising technology. To engage pediatric patient participation in the use of clinical precision medicine, receiving patient consent is required. Given the variance in consent models in pediatric medicine due to regional contexts with no standardized international guidelines, we provide a clinical primer of existing informed consent workflows and promising modern adaptations to informed consent in pediatric precision medicine. This article serves as a call to action for renewed discussion toward standardized frameworks for informed consent in pediatric patient populations.

## Introduction

Precision medicine is defined as a personalized medical approach to prevent, diagnose and treat disease using information from an individual’s genome, environment and lifestyle habits (Ginsburg and Phillips, 2018). The provision of informed consent in precision medicine demands that the patient, or in some cases their substitute decision-maker for patients without the capacity to consent, is well-informed of the treatment options and can competently assess the treatment options to authorize an optimal decision for the patient (Oberg et al., 2015).

The challenge of informed consent arises in pediatric care, where the authority for consent to make decisions in care should be considered alongside objective factors such as age and subjective factors such as the maturity of the patient, which must be determined on a case-by-case basis. Moreover, substitute decision-makers may not always make the best healthcare decisions for their child due to lack of health literacy; therefore, clinicians also play a role in protecting the child’s health from poor medical decision-making. In this perspectives article, we aim to outline modern ethical considerations and frameworks of consent-related practices in pediatric clinical precision medicine and relevant research applications.

## Informed consent frameworks

Decision-making in pediatric precision medicine is a balance of priorities between respecting the pediatric patient’s self-determination and making the most optimal decisions to improve care from the healthcare provider and surrogate decision-maker perspectives. Informed consent is defined as the voluntary, autonomous authorization of medical intervention by a patient with the capacity to understand and appreciate disclosed information relevant to the intervention (Del Carmen and Joffe, 2005). The clinical workflow of precision medicine starts with requesting informed consent from the pediatric patient or their substitute decision-makers when appropriate. Substitute decision-makers are individuals, such as parents, guardians, family members and individuals with power of attorney in pediatric medicine, with legal power to authorize healthcare-related decisions on another’s behalf if they cannot make the decisions for themselves, such as in the case of minors without knowledge and emotional understanding of the proposed intervention (Sellars et al., 2021). Decision-makers providing consent to precision medicine must assess the value of genetic screening while considering the current severity of the patient’s disease, the onset of future symptoms and the efficacy of follow-up treatment that follows from screening.

Age-based informed consent models have traditionally separated pediatric patients into two or more cohorts stratified by age, where patients below a pre-specified age threshold require joint consent to treatment from their surrogate decision-maker or assent to participate in the care plan, and patients above the threshold can provide informed consent independently (Coughlin, 2018). Beyond using age as a threshold measure of capacity to consent, the interplay between subjective factors such as patient maturity and previous ability to make medical decisions based on absolute risk should also be considered along with objective factors such as patient age to determine the patient’s holistic capacity to give informed consent for genetic testing (Salibian et al., 2018).

In the case of children incapable of providing informed consent, a common normative standard used to guide substitute decision-making is the best interests principle, where decisions are informed by the patient’s best interests (Hall et al., 2014). The best interests standard for informed consent has been challenged by proponents for an alternative framework that supposes parents have an ethical right to guide medical decisions for their children, even if they do not maximize their child’s well-being. Known as the zone of parental discretion, this ethical approach applies when parents may disagree with physicians over the recommendation for treatment and refuse to provide consent, so long as the child is not significantly harmed (Gillam, 2016).

Alternative frameworks of informed consent have focused on allowing parents to choose from reasonable options within an acceptable harm threshold or replacing substitute decision-making with supportive decision-making that establishes the patients as the primary decision-maker regardless of mental capacity (Diekema, 2004).

Pediatric patients who are not capable of consenting on their own behalf should provide assent, which allows for increased participation from pediatric participants with developing capacity to agree to proposed care. Assent is defined as the interactive process involving the disclosure of cognitively and emotionally appropriate information to the minor about the medical intervention and the voluntary agreement to choose to participate in the intervention free of undue influence (Tait and Geisser, 2017).

Assent empowers children to participate in the sharing decision-making process based on their developmental maturity and provides an opportunity for them to consider all of their options. To do so, children must be able to understand the details, benefits and risks of the procedure being performed, voluntarily decide to undergo the procedure, and communicate this choice (Rossi et al., 2003). In shared decision-making frameworks involving pediatric assent, several ethical problems and burdens remain that require further research.

First, the age by which children should provide assent depends on a case-by-case evaluation of the patient’s developmental maturity, but previous research studies have required assent from patients ranging from 6 years old (Nunes et al., 2017), 12 years old (Hein et al., 2015) and up to the age of majority based on regional legal guidelines (Coughlin, 2018). The age of medical consent varies based on local contexts; for instance, the age of medical consent is 16 years in Spain and Scotland, 18 years in Italy and France, assessed based on maturity until the age of majority in the United Kingdom and assessed based on maturity even beyond the age of majority at 18 years in Finland and Sweden (Bolcato et al., 2024). In Canada, provincial laws do not stipulate age of consent for treatment but generally deem minors (ranging from 14 to 16 years of age based on province) to have the capacity to make healthcare decisions (Coughlin, 2018).

Using age as an indicator for patient capacity has previously been challenged by opponents who suggest that pediatric patients with more early lived experiences in healthcare, such as in the case of chronic disease, may have a more mature mindset when evaluating healthcare treatment options (Miller, 2018). Second, there remains the need for education of both clinicians about the utility and application of assent as well as of patients and their substituted decision-makers about shared decision-making. Clinicians may have limited explicit knowledge of the concept of assent for medical treatment (Lee et al., 2006), motivating the need for continuing clinical education about ethical concepts to remain up-to-date about modern consent practices. For patients, age-appropriate models may be needed to help minors and their substitute decision-makers understand the concept of assent and their roles in the process (Weisleder, 2020). Maximizing the relevant, lay information necessary to understand the risk–benefit profiles of planned medical procedures coupled with novel communication mediums (Koonrungsesomboon et al., 2022), such as using age-appropriate multimedia assent documents (Wongthai et al., 2022), are one step toward addressing this need for patient education. Third, there can exist exceptional scenarios where seeking assent from children with developing capacity may be overridden if there exists a significant benefit to the child and their wellbeing would be jeopardized otherwise (Moran et al., 2011). However, we note that critiques of assent due to its heterogeneous conceptual definition may lead to the potential for harm to specific groups, such as when a minor’s dissent is ignored due to parental insistence for treatment (Wongthai et al., 2022). Loco-regional definitions of assent procedures should be consistently applied whenever possible in consultation with interdisciplinary care teams to standardize norms for assent.

Given that patient participation in precision medicine can be associated with their voluntary enrollment in related clinical trials, oversight of clinical studies by local ethics review boards remains a necessary stakeholder in the supervision of precision medicine research. The ethics review board is in the position to define the components of the informed consent processes and should ensure that such processes can be readily understood by a lay audience, such as minors and their substitute decision-makers (Matrana and Campbell, 2020). In addition, review boards play an integral role in the long-term oversight of precision medicine trial-related data governance and privacy, as defined in the terms of consent (Chen, 2020). Taken together, ethics review boards also play an emergent role in precision medicine due to the marked overlap between medical therapy and clinical research studies in precision medicine.

## Modern adaptations to improve informed consent practices

Modern consent workflows have recently been proposed to address the practical challenges to informed consent due to the unclear expectations of the benefits of precision medicine, the change in development capacity when patients reach the age of majority, and the overwhelming demands from repeated consent requests. First, having a two-step consent process where consent to genetic screening occurs one to two months following the initial consent for a diagnostic biopsy may increase the ability for parents of children to provide meaningful informed consent (Oberg et al., 2015). This two-step approach gives parents and children time and space to fully process the implications of genetic screening. Second, providing options for pediatric patients to exercise their right to re-consent for continued use of genomic data at the age of majority aligns with the patient’s right to autonomy when full informed decision-making capacity has developed (Edwards et al., 2016). Third, broad consent has previously been proposed as one way for pediatric patients and their parents to provide a one-time informed consent for the reuse of genomic data and/or health information in future research as deemed appropriate by a regulatory oversight body (Smith et al., 2016). When the mature child and parents are ready to make a decision about genetic testing for adult-onset conditions, factors such as the disease, the stage of development of the child, family dynamics and values should be considered. The conceptual development of dynamic consent frameworks in precision medicine to support both specific and broad, blanket consent is one step toward personalizing consent processes to the unique preferences of minors and their substitute decision-makers (Goncharov et al., 2022).

The age-appropriate information provided to pediatric patients and their legal representatives should, at a minimum, explain the purpose of the proposed precision medicine approach, who will have access to the genomic data, and provide clear written forms for providing informed consent (Naito et al., 2021). Genetic counseling should be provided to both the substitute decision-maker and the child about primary, clinically actionable findings and the potential for incidental findings with unknown impact to protect the patient’s right to know and not know (Naito et al., 2021). We highlight the distinction between genomic testing for evidence-based, clinically actionable findings with proven therapeutic implications compared to incidental findings, such as variants of unknown significance identified in exploratory, whole genome-scale research. Incidental findings remain commonplace in precision medicine fields such as clinical genomics, with an ongoing debate about the duty to report incidental findings based on perceived risks of disease threat (AlFayyad et al., 2021). Incidental findings may harbor unexpected results that require further high clinical suspicion for their relevance and applicability to clinical treatment, given their potential to cause patient anxiety over lack of actionability. To do so, engagement in genetic counseling, verification of results with the molecular genetics laboratory, and exploring patient perspectives over their right to know and not know remain ethical priorities (Maani et al., 2021). Despite the need for genetic counselors to navigate the complex medical and ethical considerations of pediatric precision medicine, there remains a shortage of genetic counselors and related clinicians necessary to meet the demands for precision medicine testing (Dragojlovic et al., 2020). Revisions aimed at developing more efficient contemporary genetic service models, including education to improve the competency of healthcare learners and providers in precision medicine-related tasks (Chen and Gorla, 2023), such as risk assessments and taking family histories, as well as automation of tasks through tools such as electronic decision aids and artificial intelligence, remain an ongoing area of research (Dragojlovic et al., 2020).

Notably, a review of patient perspectives related to ethical issues in precision medicine by Ahmed et al. (2023) identified several emergent themes, including data governance, patient costs, risks for discrimination, issues with the diagnostic accuracy and psychosocial implications of the findings. Future research is needed to evaluate how these ethical issues can be addressed in part through revised informed consent processes, with a particular emphasis on the need to consider pediatric patients as a growing patient population involved in precision medicine. Upon reviewing the contemporary literature on ethics in consent-related practices of pediatric precision medicine, we synthesized a list of themes that can be included as topics of discussion at the time of consent in Table 1.

**Table 1.** Discussion themes during pediatric precision medicine consent

Theme	Description
Intervention details	Information about the intervention aims and practical details, such as the healthcare provider performing the intervention
Benefits and risks	The nature, costs and anticipated benefits and risks associated with the medical intervention
Alternative options	Additional and alternative interventions in relation to the medical intervention
Scope	Roles of the different healthcare providers, substitute decision–makers and the patient in the clinical workflow
Findings	Extent to which clinically actionable and incidental findings should be discussed with the patient and used to inform selection of medical interventions
Data security and privacy	Governance policies for data access, privacy and security
Research participation	Additional consent for use of biological samples for research and future solicitation for related research studies
Resources	Contacts to ethics, review board, clinicians in care team and additional resources as appropriate
Preferences and values	Personalized preferences and values for dynamic consent as appropriate

## Conclusion

Taken together, the development of a meaningful consent process should be individualized to local contexts while seeking input from ethics review boards to ensure that legal and ethical boundaries are respected among healthcare providers, substitute decision-makers, and pediatric patients. Recent advances in consent frameworks derived from the traditional age-based consent model have proposed assessing pediatric patient’s development maturity and lived experiences in healthcare decision-making when deciding who, between the patient and their substitute decision-maker, should provide informed consent. When the substitute decision-maker is providing informed consent on behalf of a pediatric patient with developing maturity, ethical standards such as the best interests standard, harm threshold, and pediatric assent can help guide a principled, informed consent process.

Due to the complexity of pediatric precision medicine, providing ample space and counseling to make informed decisions with consideration of future medical, social and ethical implications can build rapport with patients. As the prevalence of pediatric precision medicine grows, we must develop modern ethical frameworks of informed consent involving patients, substitute decision-makers, healthcare providers and genetic counselors as part of an interdisciplinary team that collectively acts to improve the pediatric patient’s decision-making process while respecting their autonomy.

## References

[r1] Ahmed L, Constantinidou A and Chatzittofis A (2023) Patients’ perspectives related to ethical issues and risks in precision medicine: A systematic review. Frontiers in Medicine 10, 1215663. 37396896 10.3389/fmed.2023.1215663 PMC10310545

[r2] AlFayyad I, Al-Tannir M, Abu-Shaheen A and AlGhamdi S (2021) To disclose, or not to disclose? Perspectives of clinical genomics professionals toward returning incidental findings from genomic research. BMC Medical Ethics 22, 1–8. 34315465 10.1186/s12910-021-00670-y PMC8314473

[r3] Bolcato V, Franzetti C, Fassina G, Basile G, Martinez RM and Tronconi LP (2024) Comparative study on informed consent regulation in health care among Italy, France, United Kingdom, Nordic countries, Germany, and Spain. Journal of Forensic and Legal Medicine 14, 102674. 10.1016/j.jflm.2024.102674 38502996

[r4] Chen D (2020) Open data: Implications on privacy in healthcare research. Blockchain in Healthcare Today 3. 10.30953/bhty.v3.144 PMC9907398 36777059

[r5] Chen D and Gorla J (2023) The need to develop digital health competencies for medical learners. Medical Teacher 45(7), 790–791. 10.1080/0142159X.2023.2178886 36787406

[r6] Coughlin KW (2018) Medical decision-making in paediatrics: Infancy to adolescence. Paediatrics & Child Health 23(2), 138–146. 30653623 10.1093/pch/pxx127 PMC5905503

[r7] Del Carmen MG and Joffe S (2005) Informed consent for medical treatment and research: A review. The Oncologist 10(8), 636–641. 16177288 10.1634/theoncologist.10-8-636

[r8] Diekema D (2004) Parental refusals of medical treatment: The harm principle as threshold for state intervention. Theoretical Medicine and Bioethics 25, 243–264. 15637945 10.1007/s11017-004-3146-6

[r9] Dragojlovic N, Borle K, Kopac N, Ellis U, Birch P, Adam S, Friedman JM, Nisselle A, Study G, Elliott AM and Lynd LD (2020) The composition and capacity of the clinical genetics workforce in high-income countries: A scoping review. Genetics in Medicine 22(9), 1437–1449. 32576987 10.1038/s41436-020-0825-2

[r10] Edwards KL, Korngiebel DM, Pfeifer L, Goodman D, Renz A, Wenzel L, Bowen DJ and Condit CM (2016) Participant views on consent in cancer genetics research: Preparing for the precision medicine era. Journal of Community Genetics 7, 133–143. 26801345 10.1007/s12687-015-0259-8 PMC4796049

[r11] Gillam L (2016) The zone of parental discretion: An ethical tool for dealing with disagreement between parents and doctors about medical treatment for a child. Clinical Ethics 11(1), 1–8.

[r12] Ginsburg GS and Phillips KA (2018) Precision medicine: From science to value. Health Affairs 37(5), 694–701. 29733705 10.1377/hlthaff.2017.1624 PMC5989714

[r13] Goncharov L, Suominen H and Cook M (2022) Dynamic consent and personalised medicine. The Medical Journal of Australia 216(11), 547. 35611469 10.5694/mja2.51555 PMC9544476

[r14] Hall AE, Chowdhury S, Pashayan N, Hallowell N, Pharoah P and Burton H (2014) What ethical and legal principles should guide the genotyping of children as part of a personalised screening programme for common cancer?. Journal of Medical Ethics 40(3), 163–167. 23454719 10.1136/medethics-2012-101079

[r15] Hein IM, De Vries MC, Troost PW, Meynen G, Van Goudoever JB and Lindauer RJ (2015) Informed consent instead of assent is appropriate in children from the age of twelve: Policy implications of new findings on children’s competence to consent to clinical research. BMC Medical Ethics 16, 1–7. 26553304 10.1186/s12910-015-0067-z PMC4640170

[r16] Koonrungsesomboon N, Charoenkwan P, Natesirinilkul R, Fanhchaksai K, Sakuludomkan W and Morakote N (2022) What information and the extent of information to be provided in an informed assent/consent form of pediatric drug trials. BMC Medical Ethics 23(1), 113. 36384589 10.1186/s12910-022-00856-y PMC9670601

[r17] Lee KJ, Havens PL, Sato TT, Hoffman GM and Leuthner SR (2006) Assent for treatment: Clinician knowledge, attitudes, and practice. Pediatrics 118(2), 723–730. 16882829 10.1542/peds.2005-2830

[r18] Maani N, Panabaker K, McCuaig JM, Buckley K, Semotiuk K, Farncombe KM, Ainsworth P, Panchal S, Sadikovic B, Armel SR and Lin H (2021) Incidental findings from cancer next generation sequencing panels. NPJ Genomic Medicine 6(1), 63. 34282142 10.1038/s41525-021-00224-6 PMC8289933

[r19] Matrana MR and Campbell B (2020) Precision medicine and the institutional review board: Ethics and the genome. Ochsner Journal 20(1), 98–103. 32284690 10.31486/toj.19.0098 PMC7122257

[r20] Miller VA (2018) Involving youth with a chronic illness in decision-making: Highlighting the role of providers. Pediatrics 142(Supplement_3), S142–S148. 30385620 10.1542/peds.2018-0516D PMC6220652

[r21] Moran C, Thornburg CD and Barfield RC (2011) Ethical considerations for pharmacogenomic testing in pediatric clinical care and research. Pharmacogenomics 12(6), 889–895. 21692618 10.2217/pgs.10.216 PMC3164260

[r22] Naito Y, Aburatani H, Amano T, Baba E, Furukawa T, Hayashida T, Hiyama E, Ikeda S, Kanai M, Kato M and Kinoshita I (2021) Clinical practice guidance for next-generation sequencing in cancer diagnosis and treatment (edition 2.1). International Journal of Clinical Oncology 26, 233–283. 33249514 10.1007/s10147-020-01831-6 PMC7819967

[r23] Nunes LM, Ribeiro R, Niewiadonski VD, Sabino E, Yamamoto GL, Bertola DR, Gaburo N and da Silva Filho LV (2017) A new insight into CFTR allele frequency in Brazil through next generation sequencing. Pediatric Pulmonology 52(10):1300–1305. 28771972 10.1002/ppul.23774

[r24] Oberg JA, Glade Bender JL, Cohn EG, Morris M, Ruiz J, Chung WK, Appelbaum PS, Kung AL and Levine JM (2015) Overcoming challenges to meaningful informed consent for whole genome sequencing in pediatric cancer research. Pediatric Blood & Cancer 62(8), 1374–1380. 25832998 10.1002/pbc.25520

[r25] Rossi WC, Reynolds W and Nelson RM (2003) Child assent and parental permission in pediatric research. Theoretical Medicine and Bioethics 24, 131–148. 12943268 10.1023/a:1024690712019

[r26] Salibian AA, Frey JD, Choi M and Karp NS (2018) BRCA mutations in the young, high-risk female population: Genetic testing, management of prophylactic therapies, and implications for plastic surgeons. Plastic and Reconstructive Surgery 141(6), 1341–1350. 29794695 10.1097/PRS.0000000000004363

[r27] Sellars M, Tran J, Nolte L, White B, Sinclair C, Fetherstonhaugh D and Detering K (2021) Public knowledge, preferences and experiences about medical substitute decision-making: A national cross-sectional survey. BMJ Supportive & Palliative Care 14, e893–e902. 10.1136/bmjspcare-2020-002619 33722813

[r28] Smith ME, Sanderson SC, Brothers KB, Myers MF, McCormick J, Aufox S, Shrubsole MJ, Garrison NA, Mercaldo ND, Schildcrout JS and Clayton EW (2016) Conducting a large, multi-site survey about patients’ views on broad consent: Challenges and solutions. BMC Medical Research Methodology 16, 162. 27881091 10.1186/s12874-016-0263-7 PMC5122167

[r29] Tait AR and Geisser ME (2017) Development of a consensus operational definition of child assent for research. BMC Medical Ethics 18, 1–8. 28599638 10.1186/s12910-017-0199-4 PMC5466722

[r30] Weisleder P (2020) Helping them decide: A scoping review of interventions used to help minors understand the concept and process of assent. Frontiers in Pediatrics 8, 25. 32117832 10.3389/fped.2020.00025 PMC7020747

[r31] Wongthai P, Photia A, Traivaree C, Monsereenusorn C, Lertvivatpong N, Sudnawa KK and Rujkijyanont P (2022) Improving comprehension, recall and attention using multimedia‐informed assent among pediatric oncology patients: A comparative randomized controlled trial. Pediatric Blood & Cancer 69(8), e29785. 35614564 10.1002/pbc.29785

