# Supplementary material for: Ethical frameworks of informed consent in the age of pediatric precision medicine
Source: Camb Prism Precis Med. 2024 May 6;2:e6. doi: 10.1017/pcm.2024.3 (PMC11106543; doi:10.1017/pcm.2024.3)
Supplement: Peer review history

# Editor report: Recommendation: Ethical frameworks of informed consent in the age of pediatric precision medicine — R0/PR2

**DOI:** 10.1017/pcm.2024.3.pr2

**Affiliations:** University of Glasgow College of Medical Veterinary and Life Sciences, United Kingdom of Great Britain and Northern Ireland

Please specifically address the comments of Reviewers 2 and 3 only. On this occasion and with agreement of the Publisher I include Reviewer 1’s comments for full and transparent records for all, but as handling editor advise response to comments are not required.

# Editor report: Decision: Ethical frameworks of informed consent in the age of pediatric precision medicine — R0/PR3

**DOI:** 10.1017/pcm.2024.3.pr3

**Affiliations:** Institute of Cardiovascular & Medical Sciences, University of Glasgow College of Medical Veterinary and Life Sciences, United Kingdom of Great Britain and Northern Ireland

No accompanying comment.

# Editor report: Recommendation: Ethical frameworks of informed consent in the age of pediatric precision medicine — R1/PR5

**DOI:** 10.1017/pcm.2024.3.pr5

**Affiliations:** University of Glasgow College of Medical Veterinary and Life Sciences, United Kingdom of Great Britain and Northern Ireland

No accompanying comment.

# Editor report: Decision: Ethical frameworks of informed consent in the age of pediatric precision medicine — R1/PR6

**DOI:** 10.1017/pcm.2024.3.pr6

**Affiliations:** Institute of Cardiovascular & Medical Sciences, University of Glasgow College of Medical Veterinary and Life Sciences, United Kingdom of Great Britain and Northern Ireland

No accompanying comment.
